# Retraction Note: Not a passive learner but an active one: a focus on the efficacy of philosophy-based language instruction and its consequences on EFL learners’ critical thinking, engagement, and academic achievement

**DOI:** 10.1186/s40359-025-02881-z

**Published:** 2025-05-22

**Authors:** Lingxi Li, Sayed M. Ismail, Indrajit Patra, Desta Lami

**Affiliations:** 1 School of Maxism, Guangdong University of Technology, Guangzhou, 510520 China; 2 Department of English Language, College of Sciences and Humanities, Prince Sattam bin Abdulaziz University, Al-kharj, Saudi Arabia; 3 Mediterranea International Centre for Human Rights Research, Mediterranea University of Reggio Calabria, Reggio Calabria, Italy; 4 Department of English, University of Dhaka, Dhaka, Bangladesh

**Retraction Note: BMC Psychology (2024) 12:148**

10.1186/s40359-024-01648-2

The editor and the publisher have retracted this article. The article was submitted to be part of a guest-edited issue. An investigation by the publisher found a number of articles, including this one, with a number of concerns, including but not limited to compromised editorial handling and peer review process, inappropriate or irrelevant references or not being in scope of the journal or guest-edited issue. Based on the investigation’s findings the editor therefore no longer has confidence in the results and conclusions of this article.

Sayed M. Ismail disagrees with this retraction. The remaining authors have not responded to correspondence regarding this retraction.

